# Brain trauma elicits non-canonical macrophage activation states

**DOI:** 10.1186/s12974-016-0581-z

**Published:** 2016-05-24

**Authors:** Charles C. Kim, Mary C. Nakamura, Christine L. Hsieh

**Affiliations:** Department of Medicine, Division of Experimental Medicine, University of California, San Francisco, 1001 Potrero Avenue, Building 3, Room 603, Box 1234, San Francisco, CA 94143-1234 USA; Current address: Verily, Mountain View, CA 94043 USA; Department of Medicine, Division of Rheumatology, University of California, San Francisco, 4150 Clement St. 111R, San Francisco, CA 94121 USA; Research Department, Immunology Section, San Francisco VA Medical Center, 4150 Clement St. 111R, San Francisco, CA 94121 USA

**Keywords:** Innate immunity, Macrophage, Monocyte, Traumatic brain injury, Single-cell RNA sequencing, Neuroinflammation, RNA flow cytometry, Polarization, Neurotrauma, Myeloid cells

## Abstract

**Background:**

Macrophage polarization programs, commonly referred to as “classical” and “alternative” activation, are widely considered as distinct states that are exclusive of one another and are associated with different functions such as inflammation and wound healing, respectively. In a number of disease contexts, such as traumatic brain injury (TBI), macrophage polarization influences the extent of pathogenesis, and efforts are underway to eliminate pathogenic subsets. However, previous studies have not distinguished whether the simultaneous presence of both classical and alternative activation signatures represents the admixture of differentially polarized macrophages or if they have adopted a unique state characterized by components of both classical and alternative activation.

**Methods:**

We analyzed the gene expression profiles of individual monocyte-derived brain macrophages responding to TBI using single-cell RNA sequencing. RNA flow cytometry was used as another single-cell analysis technique to validate the single-cell RNA sequencing results.

**Results:**

The analysis of signature polarization genes by single-cell RNA sequencing revealed the presence of diverse activation states, including M(IL4), M(IL10), and M(LPS, IFNγ). However, the expression of a given polarization marker was no more likely than at random to predict simultaneous expression or repression of markers of another polarization program within the same cell, suggesting a lack of exclusivity in macrophage polarization states in vivo in TBI. Also unexpectedly, individual TBI macrophages simultaneously expressed high levels of signature polarization genes across two or three different polarization states and in several distinct and seemingly incompatible combinations.

**Conclusions:**

Single-cell gene expression profiling demonstrated that monocytic macrophages in TBI are not comprised of distinctly polarized subsets but are uniquely and broadly activated. TBI macrophage activation in vivo is deeply complex, with individual cells concurrently adopting both inflammatory and reparative features with a lack of exclusivity. These data provide physiologically relevant evidence that the early macrophage response to TBI is comprised of novel activation states that are discordant with the current paradigm of macrophage polarization—a key consideration for therapeutic modulation.

**Electronic supplementary material:**

The online version of this article (doi:10.1186/s12974-016-0581-z) contains supplementary material, which is available to authorized users.

## Background

Immune responses are heterogeneous and comprised of a combination of antimicrobial, tissue reparative, and regulatory functions. In certain contexts, some of these processes are pathogenic; in particular, inflammation is associated with worse outcomes in diseases ranging from cardiovascular disease to neurodegeneration. In traumatic brain injury (TBI), which accounts for 30 % of deaths related to injury [1, 2] and impacts an estimated 2 % of the US population who live with persistent disabilities resulting from TBI [1–3], neuroinflammation has been recognized as a pathogenic factor and has garnered significant attention as a potential target for therapy [1, 3–5].

Neuroinflammation develops within hours after TBI and can persist for months to years. Delivering early interventions during the first hours to days following injury may be critical for restraining lesion expansion [1, 2]. However, therapeutic amelioration of neuroinflammation is complicated by the fact that it is dynamic, multifaceted, and also critical for wound repair [1, 3, 6–8]. For example, it was shown that inhibition of CCR2 during TBI can limit lesion size; however, the same receptor is also responsible for the protection from tau pathologies [9]. As such, identification of the precise molecular pathways and cellular subsets that impact pathology and/or cognitive recovery following TBI could lead to therapies that directly antagonize harmful mechanisms while preserving, or even enriching, beneficial mechanisms.

Monocyte-derived macrophages (hereafter referred to as “macrophages”) are early responders to infection and tissue injury. Macrophages can be activated to express a variety of divergent functional programs—a process known as “polarization” [10, 11]. Both in vitro and in vivo studies demonstrate that with specific stimuli, human and mouse macrophages can polarize towards functionally divergent subsets, each possessing a distinct phenotype and gene expression profile [10–13]. Macrophage polarization and its importance for health are established in host defense, metabolism, and thermogenesis, and for pathology in obesity, cancer, allergy, and atherosclerosis [13–15]. Historically, macrophages have been classified as classical (M1) macrophages, which promote inflammation, or as alternatively activated (M2) macrophages, which restrict inflammation and foster wound repair. The M1/M2 concept has evolved to better account for the complexity of states; as such, it has been suggested that the polarization state of activated macrophages be designated by the prototypical stimulus that can produce the state in vitro—specifically, the proposed classes include M(lipopolysaccharide (LPS), interferon-γ (IFNγ)), M(IL4), M(immunocomplex (Ic)), M(IL10), and M(glucocorticoid (GC), transforming growth factor-β (TGFβ)) [11]. However, in contrast to the in vitro-derived classifications of macrophage programs, in vivo and combinatorial stimulation studies suggest that macrophages can differentiate along a spectrum of phenotypes and also exhibit plasticity in shifting from one phenotype to another [10, 11, 16]. Thus, although the definition of in vitro polarization states continues to advance, our understanding of in vivo polarization remains understudied.

Studies of macrophage polarization following TBI, including one of our own, suggest that macrophages exhibit heterogeneous expression of both inflammatory M1 and wound healing M2 markers [6–8, 17, 18]. To date, studies of macrophage activation following TBI have averaged the response by whole tissue or by bulk populations of leukocytes or macrophages. It is therefore unclear whether co-expression of M1 and M2 markers represents an admixture of differentially polarized macrophages (in the cases of whole tissue or bulk leukocyte analysis, gene expression could also be an average of microglia, macrophages, neutrophils, and other cells) or a more homogeneous, but uniquely polarized, state of macrophages. To distinguish between the above possibilities, we used RNA sequencing (RNAseq) [19] and RNA flow cytometry to analyze the polarization state of individual ipsilateral monocyte-derived brain macrophages 1 day after experimental injury. The 1-day time point was selected to understand early macrophage activation states at a time at which an immunomodulatory intervention could be delivered to minimize and/or alter the ensuing inflammatory response and associated damage. Within the TBI macrophage population, we detected gene expression signatures of M(IL4), M(LPS, IFNγ), and M(IL10) polarization; however, within individual TBI macrophages, expression of signature markers, even the highest expression of signature genes, from distinct classes commonly co-occurred within the same cell. Moreover, the expression of a given class marker was no more likely than at random to predict expression or repression of markers of another class, suggesting a lack of exclusivity in macrophage activation states. Our findings highlight the complex nature of macrophage polarization in vivo and the utility of high dimensionality, single-cell assays for its interrogation.

## Methods

### Animals

Twelve- to sixteen-week-old male C57BL/6 cage mate mice (Jackson Laboratories, Sacramento, CA) were housed at the San Francisco VA Medical Center. Controlled cortical impact (CCI) or sham surgery was performed as approved by the VA Animal Care Committee. Animals were anesthetized with 3 % isoflurane with oxygen and were administered bupivacaine s.c. above the skull. The scalp was incised. A 2-mm circular craniectomy with center coordinates of 1.5 mm lateral and 2.3 mm posterior to the bregma point was performed. Care was taken to not breach the dura by not drilling completely through the skull. No animals in this study showed signs of excessive bleeding or dural breach from the craniectomy procedure. TBI was induced by a sterile, pneumatic, circular, flat-tipped piston with impact parameters of 3 m/s velocity, 150 ms dwell time, and 1.5 mm depth (Amscien Instruments, Richmond, VA, with extensive modifications by H&R Machine, Capay, CA). These CCI parameters led to bleeding, and pressure was gently applied with a cotton swab until bleeding had halted, and the skin was stapled shut. Sham-injured mice received surgical procedures without piston impact.

### Brain leukocyte isolation

Ipsilateral brain hemispheres were harvested 1 day post-surgery following whole-body perfusion to eliminate circulating blood. Tissues were pooled, mechanically dissociated into suspension, and washed in GKN buffer (8 g/L NaCl, 0.4 g/L KCl, 1.41 g/L Na_2_HPO4, 0.6 g/L NaH_2_PO4, and 2 g/L D(+) glucose, pH 7.4). Cells were resuspended in NOSE buffer (4 g/L MgCl_2_, 2.55 g/L CaCl_2_, 3.73 g/L KCl, 8.95 g/L NaCl, pH 6–7) supplemented with 200 U/ml DNase I (Sigma-Aldrich, St. Louis, MO) and 0.2 mg/ml collagenase type I (Worthington Biochemical, Lakewood, NJ) and incubated at 37 °C for 30 min. Washed cells were separated on a discontinuous isotonic Percoll gradient (90 % Percoll, 10 % 1.5 M NaCl, GE Biosciences, Pittsburgh, PA) by suspending cells in 20 mL of a 1.03 g/ml Percoll solution in GKN buffer and underlaying the cells with 10 ml of 1.095 g/L Percoll in PBS. Cells were centrifuged at 900×*g* for 20 min without brake. The buffy layer was isolated for further study.

### Bone marrow-derived macrophages

Mice were sacrificed by inhaled isoflurane sedation followed by cervical dislocation. Femurs were harvested, bone ends were snipped, and the bones were flushed with PBS. Marrow was resuspended and spun into a pellet. Cells were resuspended into 2 ml of red blood cell lysis buffer for 2 min and washed with PBS. Cells were plated into two 10-cm non-TC-coated petri dishes per femur. Bone marrow cells were cultured in RPMI or alpha-MEM (Thermo Fisher Scientific, Waltham, MA) supplemented with 10 % FCS, 1 % penicillin-streptomycin, and 10 % CMG for 8 days. Bone marrow-derived macrophages (BMDM) were polarized with 20 ng/ml IL-4 (Peprotech, Rocky Hill, NJ) for 18 h or 100 μg/ml LPS from *Salmonella enterica* (Sigma-Aldrich) for 2–3 h.

### Single-cell RNA sequencing

Ipsilateral TBI brain hemispheres were pooled from three age-matched male cage mate mice and brain leukocytes were isolated as described above. TBI brain macrophages were sorted to 99.8 % purity on a FACSAria IIu (BD Biosciences, San Jose, CA) located at the San Francisco General Hospital. The following antibodies from eBioscience (San Diego, CA) were used: CD45 (clone 30-F11), Ly6G (clone 1A8), F4/80 (clone BM8), and CD11b (clone M1/70). Sytox blue (Thermo Fisher Scientific) was used at a final concentration of 1 μm. Following cell sorting, single cells were immediately loaded onto a Fluidigm C1 chip (Fluidigm Corporation, South San Francisco, CA) and prepared into RNAseq libraries following manufacturer protocols. Nextera XT (Illumina, San Diego, CA) DNA library generation reagents were used to fragment and barcode libraries. Single-cell DNA libraries were sequenced as 51 bp reads on an Illumina HiSeq 2500 running in high output mode by the UCSF Center for Advanced Technology. Reads were aligned to the GRCm38 release of the mouse genome using RSEM with default parameters. Gene expression was reported as transcripts per million (TPM). Quality analysis was performed using custom scripts and FASTQC (http://www.bioinformatics.babraham.ac.uk/projects/fastqc/). Samples with fewer than one million aligned reads or with fewer than 50 % of reads mapped were excluded.

### RNA flow cytometry

Intracellular RNA flow cytometry was performed using PrimeFlow RNA (Affymetrix, Santa Clara, CA) reagents following manufacturer’s protocols. Cell viability was assessed by Fixable Viability dyes eFluor 506 or eFluor 455UV (eBioscience). Cell surface markers were stained using antibodies against CD45 (clone 30-F11), CD11b (clone M1/70), Ly6G (clone 1A8), and Ly6C (clone HK1.4). A *DapB* RNA probe, a probe for RNA of a bacterial gene, served as a control for non-specific nucleic acid binding that could occur in activated macrophages. The following RNA probes were used: *DapB*, *Arg1*, *Mrc1*, *Chi3l3*, *Tnf*, and *Il1b*. Cell staining was analyzed on a FACSAria IIu located at the San Francisco VA Medical Center or the San Francisco General Hospital. Data analysis was performed by using FlowJoX (Treestar, Ashland, OR). For BMDM, three independent RNA flow cytometry experiments were performed. For TBI mice, six separate RNA flow cytometry experiments were performed, each with pooled ipsilateral hemispheres from eight age-matched cage mates. For sham-injured mice, three independent RNA flow cytometry experiments were performed, with pooled ipsilateral hemispheres from six to ten age-matched cage mates.

### Statistical analysis

Prism 6.0 (Graphpad, San Diego, CA) software was used to perform linear regression analyses and Mann-Whitney U tests.

## Results

### Validation of RNAseq profiles of single monocytes responding to TBI

Our previous approach of bulk-profiling-purified brain macrophages responding to experimental TBI demonstrated that macrophages expressed a mixture of signature polarization genes representing both classical and alternative activation [6]. It was unclear whether this mixed polarization signature reflected (in a non-mutually exclusive manner): (1) a mixture of classically and alternatively activated macrophage subsets, (2) an intermediate polarization state of macrophages that are transitioning between states, or (3) unusual subsets of macrophages adopting a stable state distinct from our current definitions of polarization. We thus employed single-cell RNA sequencing to profile the whole transcriptome at the resolution of individual cells.

**Fig. 1:**
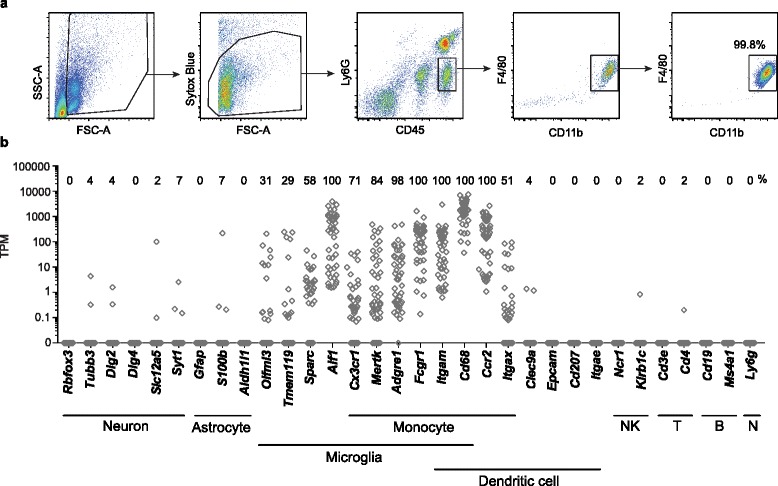
Transcriptional profiles of lineage markers as determined by single-cell RNA sequencing of individual TBI macrophages. **a** Mouse leukocytes were harvested from pooled ipsilateral hemispheres of brain tissue 1 day post-TBI and cell sorted for macrophages by flow cytometry. Flow cytometry gates for cell sorting of live TBI macrophages (CD45^hi^ Ly6G^−^ CD11b^+^ F4/80^+^) are shown. TBI macrophages were sorted to high purity and processed for single-cell RNA sequencing. **b** Transcriptomes of 45 individual TBI brain macrophages were analyzed for cell lineage marker expression. Each *diamond symbol* in the stacked dot plot represents a single TBI macrophage. For each cell, gene expression of markers of neurons, astrocytes, microglia, monocytes, dendritic cells, NK cells, T and B cells, and neutrophils (*N*) is shown as transcripts per million (TPM). The percentage of TBI brain macrophages positively expressing a gene (TPM > 0.1) is reported above each column

We purified individual macrophages isolated from the ipsilateral hemisphere of mouse TBI brains 1 day post-injury. Monocyte-derived macrophages of hematopoietic origin were defined as CD45^hi^ Ly6G^−^ CD11b^+^ F4/80^+^ cells (Fig. 1a) [20], which we demonstrated are a population of infiltrating macrophages that fail to increase in *Ccr2*^*−/−*^ mice 1 day after TBI [21]. Macrophages were isolated to high purity (99.8 %) by two successive rounds of flow cytometric sorting (Fig. 1a), and single purified cells were isolated using a Fluidigm C1 and processed into individual RNAseq libraries for transcriptome analysis. Libraries for 63 cells were prepared and sequenced; average base quality across all samples was >Q36, and reads exhibited other characteristics of high-quality sequence (Additional file 1: Figure S1). Upon alignment of reads to the mouse genome, 45 cells exceeded our minimum quality criteria.

To validate the lineage of our sorted cells, we assessed the expression of 32 markers of both non-hematopoietically and hematopoietically derived cells. Expression of the lineage markers was consistent with high purity of the isolated macrophages. Specifically, all cells expressed high levels of myeloid markers, such as *Fcgr1* (CD64) and *Cd68*, and nearly every cell expressed adhesion G protein-coupled receptor E1 (*Adgre1;* F4/80) and mer proto-oncogene, tyrosine kinase (*Mertk*) (Fig. 1b). These static measurements exhibited variation in mRNA levels over 4 to 5 orders of magnitude but included genes whose protein products were used in our flow cytometry gating strategy, such as *Adgre1* (F4/80) and *Itgam* (CD11b), in which cases the protein level was relatively uniform as measured by flow cytometry (<1 order of magnitude; Fig. 1a). Additional myeloid-associated markers were expressed in all, or most of these cells included integrin, alpha M (*Itgam;* CD11b), and C-X_3_-C chemokine receptor 1 (*Cx3cr1*). The lack of detected expression for any of the principal monocyte markers was not related to sequence undersampling, as cells with zero expression of a given marker were not associated with low read coverage (Additional file 2: Figure S2). Two markers commonly associated with both microglia and infiltrating macrophages—*Aif1* (Iba1) and *Sparc*—were detectably expressed on all of the cells and about half of the cells, respectively (Fig. 1b). Although microglia are often indistinguishable from macrophages based on expression of commonly used markers, all cells analyzed expressed high levels of *Ccr2* transcript, which is not expressed by microglia [22]. Furthermore, by flow cytometry, the microglia as a bulk population were distinguishable by their lower levels of CD45 expression (Fig. 5c). The vast majority of the cells expressed undetectable levels of genes that are markers for neurons, astrocytes, and other leukocyte lineages including dendritic cells, NK cells, T cells, B cells, and neutrophils (Fig. 1b).

### TBI macrophages express mixed polarization signatures

**Fig. 2:**
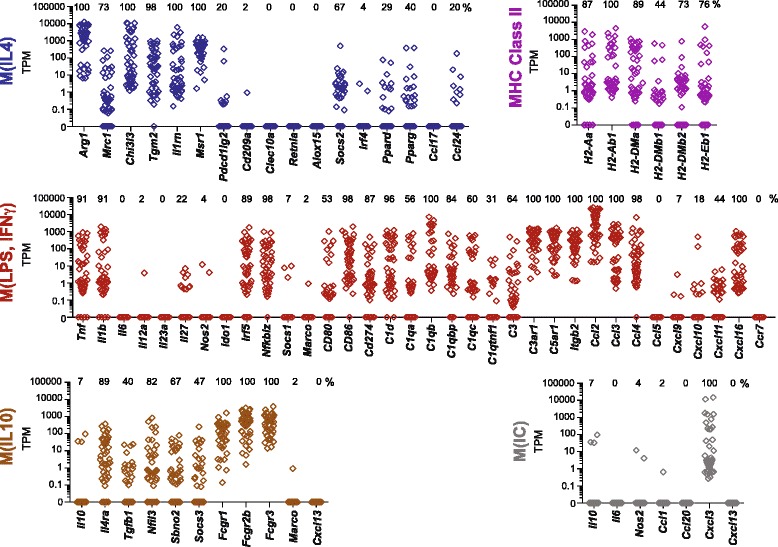
TBI brain macrophages express several signature genes of M(IL4), M(IL10), and M(LPS, IFNγ) macrophage classes. Gene expression of 74 macrophage polarization genes representing distinct macrophage classes of M(IL4), M(LPS, IFNγ), M(IL10), and M(IC) were analyzed in individual TBI macrophages by single-cell RNA sequencing. Each *diamond symbol* in the stacked dot plot represents a single TBI macrophage, 45 individual cells were analyzed. The percentage of TBI brain macrophages positively expressing a gene (TPM > 0.1) is reported above each gene column

Of the 38,126 annotated genes, 6150 genes (16.1 % of all genes) were detectably expressed (TPM > 0.1 in >50 % of cells). Hierarchical clustering has been used to identify population structure within heterogeneous tissues [23–25]; similar analysis of the whole macrophage transcriptomes did not identify any apparent subsets. For example, dimensionality reduction using principal component analysis (PCA) did not identify sub-groups of macrophages when applied to the whole transcriptome or when applied to the top 10 % of genes exhibiting the most variance across individual cells (Additional file 3: Figure S3). This may be due, at least in part, to our profiling of a comparatively homogeneous subset. Thus, we focused our subsequent analysis on 74 genes that have been verified as markers of mouse macrophage subsets and polarization [12, 13, 26–28], including a set of 26 genes proposed as a consensus of markers of M(IL4), M(LPS, IFNγ), M(IL10), and M(IC) polarization (Fig. 2) [11].

Overall, numerous genes representing M(IL4), M(LPS, IFNγ), and M(IL10) groups were moderately to highly expressed in all or many TBI macrophages, and one gene representing the M(IC) group was highly expressed by all cells (Fig. 2). We observed a wide range in the magnitude of M(IL4) marker expression, with particularly high levels of *Arg1* in all cells; the large majority of the cells (93 %) expressed *Arg1* at high levels (TPM ≥ 10). There were modest levels of the alternative activation marker chitinase 3-like 3 (*Chi3l3*) mRNA in all cells*.* Mannose receptor, C-type lectin 1 (*Mrc1*), and suppressor of cytokine signaling-2 (*Socs2*) were each expressed by ~70 % of the cells, but they were not always expressed by the same cell as less than half of the cells co-expressed both markers (TPM > 0.1) (Fig. 4a). Less well-described M(IL4) markers in mice, transglutaminase 2 (*Tgm2*) and interleukin 1 receptor antagonist (*Il1rn*), both of which are noted to be M(IL4) markers in humans [11, 26, 28], were expressed by all or nearly all cells (Fig. 2). Macrophage scavenger receptor 1 (*Msr1*) was also expressed by all cells (Fig. 2).

With regard to M(LPS, IFNγ) genes, interleukin 1 beta (*Il1b)* was detectably expressed in 91 % of the cells (TPM > 0.1), some of which exhibited high levels of expression (29 % with TPM > 10) (Fig. 2). Similar observations were made for *Tnf* (91 % cells with TPM > 0.1, 38 % with TPM > 10) (Fig. 2). Over 90 % of cells highly expressed one or more markers of M(LPS, IFNγ) polarization, such as interferon regulatory factor 5 (*Irf5*) or nuclear factor of kappa light polypeptide gene enhancer in B cell inhibitor, zeta (*Nfkbiz*) (Fig. 2). Several genes associated with antigen presentation, complement, and chemotaxis were also expressed by all or many TBI macrophages, including *Cd86*; complement component 1, q subcomponent, beta polypeptide (*C1qb*); complement component 3a receptor 1 (*C3ar1*); complement component 5a receptor 1 (*C5ar1*); C-C chemokine ligand 2 (*Ccl2*); *Ccl3*; *Ccl4*; and *Cxcl16* (Fig. 2). Although they are not markers of macrophage polarization, other chemotaxis molecules, *Ccl6, Ccl7, Ccl12, Cxcl2, Cxcr2*, *Ccr1, Ccr2, Ccr5,* and *Cxcr4*, were highly expressed by nearly all cells (Additional file 4: Figure S4). Multiple major histocompatibility class II (MHCII) genes were expressed on the majority of cells (*H2-Aa, H2-DMa, H2-DMb2, H2-Eb1*) or all cells (*H2-Ab1*) (Fig. 2), although MHCII molecules characterizes both M(IL4) and M(LPS, IFNγ) cells [12, 26].

Four M(IL10) markers—interleukin 4 receptor, alpha (*Il4ra*); nuclear factor, interleukin 3, regulated (*Nfil3*); strawberry notch homologue 2 (*Sbno2*); and suppressor of cytokine signaling 3 (*Socs3*)—were expressed by TBI macrophages at frequencies of 91, 82, 69, and 47 %, respectively. M(IL10)-associated Fc receptors for IgG, *Fcgr1* (CD64), *Fcgr2b* (CD32), and *Fcgr3* (CD16) were expressed on all cells (Fig. 2), although *Fcgr1* has also been suggested to be a marker of monocyte ontogeny [29], in addition to polarization [12, 26]. The data also revealed minimal expression of signatures associated with M(IC) polarization, with the exception that all the cells expressed *Cxcl3* [26] (Fig. 2). Thus, the macrophage response to TBI is highly heterogeneous, with broad expression of markers from each polarization class, and certain markers from each class were expressed by all cells.

**Fig. 3:**
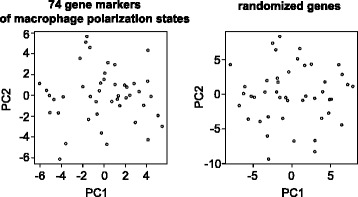
PCA of macrophage polarization gene expression is compared to PCA of randomized genes. PCA of 74 markers of macrophage polarization (*left*) and PCA of the same expression data in randomized order (*right*) are shown for comparison

PCA was used to determine relationships and patterns among the 74 polarization markers. However, the macrophage polarization genes were not coherently expressed as their relationships of expression were no more related than data in which the order of expression values were randomized (Fig. 3).

### Complexity and incoherence in polarization signature gene expression

**Fig. 4:**
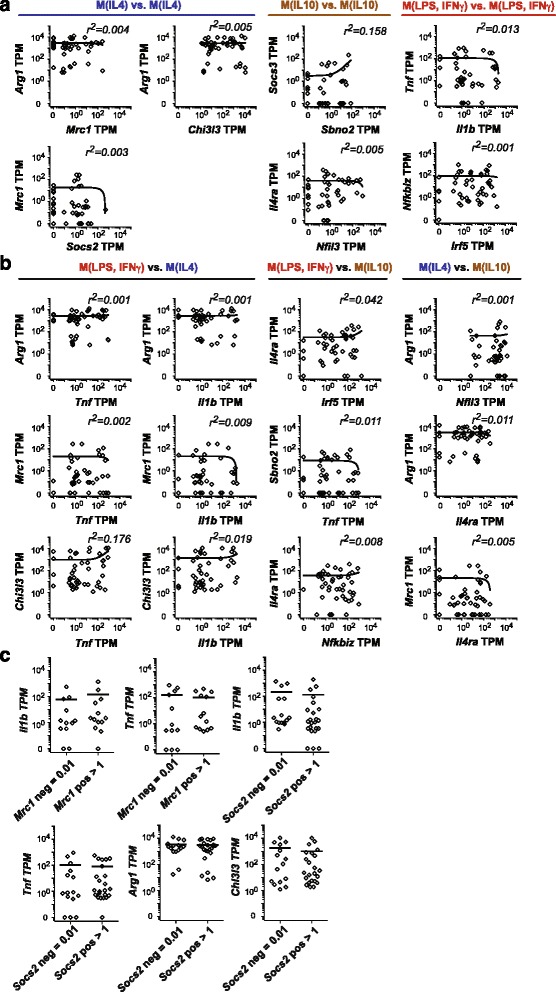
Co-expression analysis of macrophage polarization markers demonstrate incoherent expression in TBI macrophages. **a** Pairwise dot plots of absolute expression values of widely accepted signature macrophage polarization markers in TBI macrophages are shown. Co-expression analyses of signature genes within the same group were analyzed by linear regression analysis, and Pearson’s correlation coefficients (*r* ^2^) and the lines of best fit are presented. **b** Pairwise dot plot of absolute expression values of signature macrophage polarization markers across classes were analyzed in TBI macrophages. Results of linear regression analysis and *r* ^2^ are shown. **c** Signature macrophage polarization markers were analyzed for their capacity to predict gene expression of other signature macrophage polarization markers and shown here in stacked dot plots. The capacity of *Mrc1* (TPM > 1) or lack of *Mrc1* (TPM = 0.01) in a TBI macrophage to predict the mean expression level of *Il1b* or *Tnf* was statistically analyzed by Mann-Whitney U tests. All *p* values were insignificant (*p* < 0.05). Similarly, the expression of or lack of expression of *Socs2* in a TBI macrophage failed to predict the mean expression level of *Tnf*, *Arg1*, and *Chi3l3*

In order to more closely examine whether individual TBI macrophages exhibited signatures of distinct states of polarization, we more closely assessed co-expression of broadly accepted markers of mouse macrophage polarization [11]. Correlation between gene expression within the same polarization group was assessed by linear regression (Fig. 4a). As described above, every cell expressed *Arg1* and *Chi3l3* at moderate to high levels, but there was no correlation of the expression level of the two genes (*r*^2^ = 0.0005) (Fig. 4a). The expression of *Arg1* and *Mrc1* was also unrelated (*r*^2^ = 0.004) (Fig. 4a). The correlation between genes of the M(IL10) group, *Sbno2*:*Socs3* and *Nfil3:Il4ra*, were similarly poor (Fig. 4a). Gene pairs of the M(LPS, IFNγ) group, *Il1b:Tnf* and *Irf5:Nfkbiz*, had low coefficients of determination of 0.013 and 0.001, respectively.

Expression of polarization markers across groups also lacked collinearity. Notably, all of the cells expressing *Tnf* by RNAseq also expressed both *Arg1* and *Chi3l3*, with no apparent relationship between the expression of *Tnf* with either alternative activation marker (Fig. 4b, *Tnf*:*Arg1 r*^2^ = 0.001, *Tnf*:*Chi3l3 r*^2^ = 0.176). *Il1b* expression exhibited similar characteristics with no correlation with either M(IL4) markers (*Il1b*:*Arg1 r*^2^ = 0.001, *Il1b*:*Chi3l3 r*^2^ = 0.019) (Fig. 4b). We also compared M(LPS, IFNγ) markers with expression of a less strongly expressed alternative activation marker, *Mrc1*, which we hypothesized might reveal more underlying structure. This was not the case; *Mrc1* subsets were no less likely to express *Il1b* or *Tnf* (Fig. 4b, *Tnf*:*Mrc1 r*^2^ = 0.002, *Il1b*:*Mrc1 r*^2^ = 0.002). Analysis of co-expression of M(LPS, IFNγ) genes vs M(IL10) genes, such as *Irf5*:*Il4ra*, *Tnf*:*Sbno2*, and *Nfkbiz*:*Il4ra*, also showed no relationship (*r*^2^ = 0.008–0.042) (Fig. 4b). Finally, M(IL10) vs M(IL4) gene expression pairs, including *Nfil3*:*Arg1*, *Il4ra*:*Arg1*, and *Il4ra*:*Mrc1*, lacked correlation (*r*^2^ = 0.001–0.011) (Fig. 4b).

To further determine if TBI macrophages exhibit coherent expression of polarization markers, we analyzed whether expression of widely used polarization markers of classical or alternative activation could predict expression or repression of other polarization markers. For each gene, cells were divided into either cells expressing the gene (TPM > 1) or cells not expressing the gene (TPM = 0.01). *Mrc1*^*+*^ TBI macrophages expressed comparable levels of *Il1b* and *Tnf* as compared to *Mrc1*^*−*^ cells (Fig. 4c); similarly, *Socs2*^*+*^ TBI macrophages had similar mean levels of co-expression of *Il1b*, *Tnf*, *Arg1*, and *Chi3l3* when compared to *Socs2*^*−*^ cells (Fig. 4c). In conclusion, we did not observe coherence in the expression of key polarization markers with one another, even within the same polarization class.

### TBI macrophages co-express in vitro-defined polarization markers in complex combinations

It has been shown in vitro that polarization to one activation program inhibits the gene expression of other polarization programs [10]. In the above analysis of gene co-expression, we found that a number of individual macrophages co-expressed the highest expression levels of signature genes representing distinct classes in seemingly incompatible combinations. For example, the TBI macrophages that expressed the highest levels of *Arg1* (TPM > 100) were also among the cells that expressed the highest levels of *Tnf* or *Il1b* (TPM > 100) (Fig. 4b). Similarly, among the cells that expressed the highest levels of *Arg1* (TPM > 100) were cells that expressed the highest levels of M(IL10) signature genes *Nfil3* or *Il4ra* (TPM > 100) (Fig. 4b). The cells that expressed the highest levels of M(LPS, IFNγ) markers, such as *Irf5* or *Nfkbiz*, often expressed the highest levels of *Il4ra* (Fig. 4b).

Moreover, we consistently observed that individual TBI macrophages commonly exhibited simultaneous and strong co-expression genes generally found to be inversely expressed in vitro (Fig. 4b). For example, we observed high frequencies of *Arg1*^*hi*^*Tnf*^*hi*^ cells, *Chi3l3*^*hi*^*Ilb*^*hi*^ cells, *Il4ra*^*hi*^*Nfkbiz*^*hi*^ cells, and *Arg1*^*hi*^*Il4ra*^*hi*^ cells, among others. The percentage of cells with very high co-expression (TPM > 100) of at least two genes, each from a distinct polarization group, was ~55 % (25/45 cells). Eleven percent of cells (5/45) showed high co-expression of at least three genes (TPM > 100), each one from a different polarization group, such as *Arg1*^*hi*^*Tnf*^*hi*^*Nfil3*^*hi*^ and *Arg1*^*hi*^*Irf5*^*hi*^*Nfkbiz*^*hi*^*Il4ra*^*hi*^ cells. Thus, not only can individual macrophages adopt a state representing multiple distinct activation programs, but they can do so in a variety of combinations that cross the boundaries of in vitro polarization states.

### Validation of mixed TBI macrophage activation programs by RNA flow cytometry

**Fig. 5:**
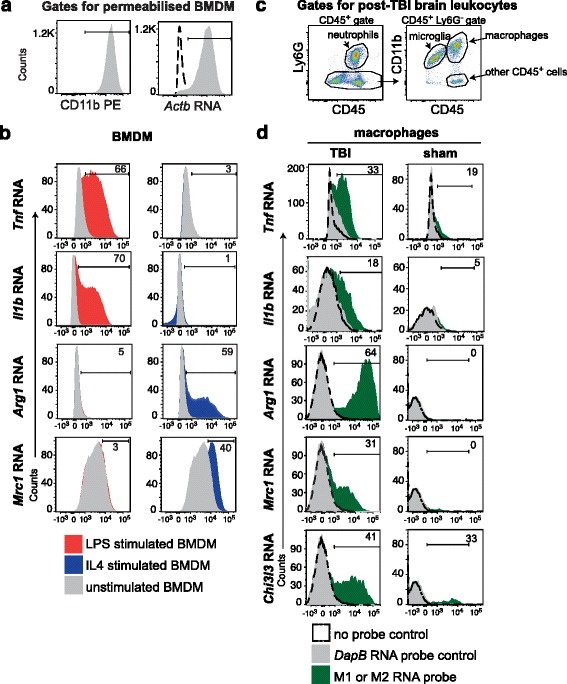
Signature polarization genes are upregulated in brain macrophages post-TBI. **a** RNA flow cytometry was performed on bone marrow-derived macrophages (BMDM) that were gated for surface *Itgam* (CD11b) expression and intracellular *Actb* RNA expression. The latter is a gate for permeabilized cells. **b** RNA flow cytometry was performed on unstimulated (*gray*), LPS-polarized (*red*), and IL4-polarized (*blue*) BMDM. Probes for RNA expression of M(LPS, IFNγ) markers, *Tnf* and *Il1b*, and M(IL4) markers, *Arg1* and *Mrc1* were used to stain permeabilized BMDM and analyzed by flow cytometry. *N* = 3 independent experiments. **c** Ipsilateral hemisphere brain leukocytes were harvested from mice 1 day after TBI or sham surgery. Flow cytometry plots represent the gates used for live macrophages. **d** Macrophages harvested from ipsilateral brain hemispheres of mice 1 day after TBI (left, *n* = 6 independent experiments) and sham surgery (right, *n* = 3 independent experiments) were assessed by RNA flow cytometry. Analysis markers were drawn based on non-specific background staining using *DapB* RNA probes for a bacterial gene. The percentage of positive expression shown is the difference in the percent of brain macrophages expressing the M1 or M2 gene and the percent of cells with background detection of DapB RNA

To corroborate the RNAseq finding that individual macrophages co-express high levels of signature polarization genes across polarization classes, we used RNA flow cytometry. First, probe specificity was confirmed using BMDM polarized in vitro. Permeabilized BMDM were gated for their expression of *Itgam* (CD11b) and *Actb* (β-actin) RNA (Fig. 5a). Positive expression was determined by comparison with a non-specific RNA probe, *DapB*. In LPS-stimulated BMDM, RNA probes detected expression of *Tnf* and *Il1b*, with no detection of *Arg1* or *Mrc1* above unstimulated controls (Fig. 5b). In IL4-stimulated BMDM, RNA flow cytometry detected *Arg1* and *Mrc1* expression, with no upregulation of M(LPS, IFNγ) markers (Fig. 5b).

**Fig. 6:**
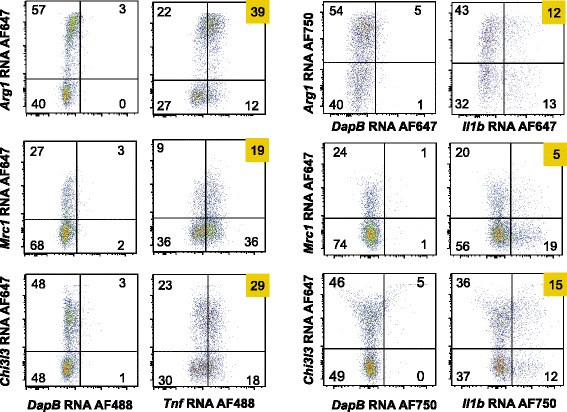
RNA flow cytometry validates that TBI macrophages co-express macrophage polarization markers across classes in unusual combinations. RNA flow cytometry for M(LPS, IFNγ) markers, *Tnf* and *Il1b*, were analyzed for their co-expression with M(IL4) markers, *Arg1*, *Mrc1*, and *Chi3l3*, in ipsilateral TBI brain hemispheres 1 day post-TBI. Quadrant gates were drawn based on DapB RNA probe binding. *N* = 3 independent experiments, with pooled tissues of eight age-matched cage mate mice for each experiment

We next performed RNA flow cytometry on TBI brain leukocytes, as well as on sham-injured brain leukocytes, isolated from the ipsilateral hemisphere 1 day after surgery (Fig. 5c, d). TBI macrophages exhibited upregulation of *Tnf*, *Il1b*, *Arg1*, *Mrc1*, and *Chi3l3*, compared to macrophages from sham-injured brains (Fig. 5d). Compared to RNAseq, the overall sensitivity of RNA flow cytometry was lower, as expected. As observed by RNAseq, RNA flow identified macrophage subsets exhibiting high expression of *Tnf* in conjunction with M(IL4) gene expression (i.e., *Arg1*, *Mrc1*, or *Chi3l3*) (Fig. 6). Similar observations were made for *Il1b* and its co-expression with *Arg1*, *Mrc1*, or *Chi3l3* (Fig. 6). These data corroborated the existence of macrophages co-expressing M(LPS, IFNγ) genes with M(IL4) genes at the highest levels.

## Discussion

We previously reported mixtures of signature polarization genes in macrophages responding to experimental TBI [6]. To elucidate whether the mixed polarization of macrophages detected in TBI was due to the presence of a mixture of polarized subsets and/or whether novel states of macrophage activation existed, we employed two powerful assays for elucidating transcript levels at the resolution of single cells: single-cell RNAseq and RNA flow cytometry. Using these approaches, we showed that TBI macrophages not only express several signature genes of distinct polarization states (i.e., M(IL4), M(LPS, IFNγ) and M(IL10)) but that signatures from distinct states are often expressed in combination with one another simultaneously in a single cell. Although demonstrating the functional relevance of these states will require further study, protein analysis by histology reported by others showed expression of Il1b and tumor necrosis factor (TNF) segregated and colocalized in macrophages in ischemic brain injury [30]; in TBI, histological protein analysis revealed Arg1^+^iNOS^*+*^ microglia/macrophages in the brain tissue [4] as well as CD36 and MARCO co-expression on microglia/macrophages [18]. Future efforts should more deeply profile protein expression with a larger number of parameters and use perturbational approaches to determine the associated cellular functions of these understudied leukocyte subsets. Our data extend earlier work to demonstrate that TBI macrophages exhibit expression of signature genes from two or more polarization states in a broad range of unexpected gene combinations. Further, these findings provide evidence that the mixed macrophage response to TBI is an unusual activation state rather than admixture of differentially polarized macrophages. Importantly, our data highlight the complex nature of macrophage activation in vivo that is inadequately described by current nomenclature based on in vitro studies.

In fact, we did not detect any TBI macrophages that were distinctly polarized to a single state; all macrophages exhibited expression of markers associated with two or more known polarization states. Given that macrophages are known to have some limited plasticity to transition from one polarization state to another, we considered that TBI macrophages could be expressing mixed polarization markers due to being in a state of transition. Although we cannot completely rule out the possibility that TBI macrophages at this time point are transitioning to distinct polarization states, one would expect an assay of single cells to reveal a distribution of cells more strongly polarized to one or another state, with lower mean levels of gene expression of the associated genes during the transition. We did not observe this to be the case; many cells with the highest expression levels of a signature of one polarization state also exhibited the highest expression levels for a distinct polarization state. The determination of gene expression profiles at downstream time points is needed to better elucidate the full repertoire and dynamics of macrophage activation states over the course of TBI. Further studies to investigate and compare the pathways of activation for the tissue-resident macrophages in the brain, the microglia, may shed light on how cellular origin affects function and how to more precisely target therapeutics.

The above findings have important clinical implications. Several chemotaxis and complement-associated genes were expressed by all TBI macrophages, including *Ccr2*. These may be potential therapeutic targets or biomarkers. In fact, studies have identified *Ccr2* as a potential target to improve the functional outcomes of TBI [5, 7, 21, 31]. Taken together, these data suggest that individual *Ccr2*-dependent TBI macrophages are activated to become multi-dimensional, but that their net influence is debilitating. Most notably, our findings indicate that the same macrophages responsible for pathological neuroinflammation also express genes that promote immunoregulation and wound healing. By extension, it may be beneficial to focus therapeutic efforts on modulating myeloid cell activation rather than depleting these cells or completely blocking their infiltration. However, our studies show that efforts to shape TBI macrophages must address the challenge that the target cells are already broadly activated.

## Conclusions

Traumatic brain injury (TBI) is a major public health problem with no known effective pharmacological therapy. Primary goals of therapy are to preserve brain tissue and to promote wound healing, which might be possible through modulation of the neuroinflammatory response. Using single-cell gene expression methods, we show that monocyte-derived macrophages, a major population of innate immune cells responding to acute TBI, are not comprised of distinctly polarized pro-inflammatory or pro-reparative subsets. Instead, there is a deep complexity to acute TBI macrophage activation in vivo, with cells adopting unique activation states and concurrently adopting features associated with both inflammation and wound healing, thus shifting the paradigm of in vivo macrophage polarization. These data reveal important considerations for therapeutic approaches that aim to alter inflammation in vivo and shape macrophage activation.

### Ethics approval

All animal studies were performed under a protocol approved by the San Francisco VA Medical Center Animal Care Committee.

### Consent for publication

Not applicable.

### Availability of data and materials

The RNA sequencing data has been submitted to the Gene Expression Omnibus data repository and can be accessed through the following link: http://www.ncbi.nlm.nih.gov/geo/query/acc.cgi?acc=GSE79510.

## Additional files

Additional file 1: Figure S1. Quality of RNA sequencing data. (a) Average base quality (Q score) per sample. (b) Average GC composition (%) of reads per sample. (c) Average mapped read count per sample. (PDF 372 kb) Additional file 2: Figure S2. Expression of myeloid lineage genes is not associated with read coverage. Expression (log_10_ TPM) as a function of mapped read coverage does not reveal bias. Of particular note, cells with zero detected expression of a given marker do not correlate with read coverage. (PDF 283 kb) Additional file 3: Figure S3. Dimensionality reduction analysis of the whole transcriptome or of the top 10 % variable genes across all individual TBI macrophages shows a lack of coherent population structure*.* (a) PCA analysis of the whole transcriptome (38,126 genes). The same analysis applied to the same data, but in randomized order, is shown for comparison. (b) PCA analysis of the top 10 % of genes with the highest variance (3815 genes). Expression data from the same 3815 genes were randomly ordered and are shown for comparison. (PDF 166 kb) Additional file 4: Figure S4. Gene expression analysis of chemotaxis genes not associated with macrophage polarization. All or nearly all TBI macrophages express moderate to high levels of several chemotaxis-associated genes as shown. Each diamond symbol represents one TBI macrophage. The percentage of cells positively expressing a gene is shown above each gene’s column. (PDF 160 kb)

## Abbreviations

BMDM: bone marrow-derived macrophages
CCI: controlled cortical impact
GC: glucocorticoid
Ic: immunocomplex
IFNγ: interferon-γ
IL1b: interleukin 1 beta
LPS: lipopolysaccharide
PCA: principal component analysis
RNAseq: RNA sequencing
TBI: traumatic brain injury
TGFβ: transforming growth factor-β
TNF: tumor necrosis factor
TPM: transcripts per million
