# Supplementary figures and images for: Brain trauma elicits non-canonical macrophage activation states
Source: J Neuroinflammation. 2016 May 24;13:117. doi: 10.1186/s12974-016-0581-z (PMC4879757; doi:10.1186/s12974-016-0581-z)

**Figure S1**

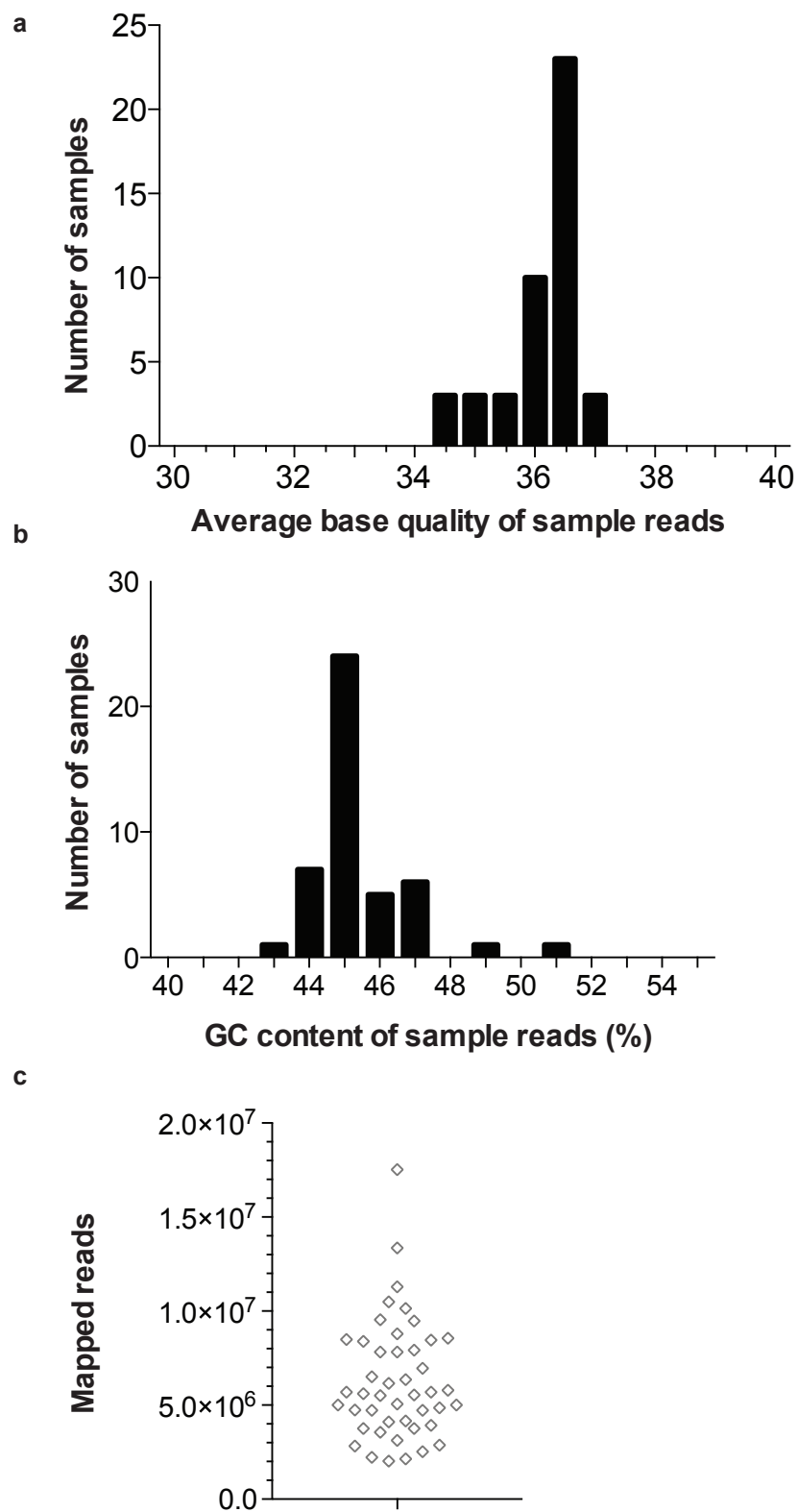

Supplement: Additional file 1: Figure S1. — Quality of RNA sequencing data. (a) Average base quality (Q score) per sample. (b) Average GC composition (%) of reads per sample. (c) Average mapped read count per sample. (PDF 372 kb) [file 12974_2016_581_MOESM1_ESM.pdf]

**Figure S2**

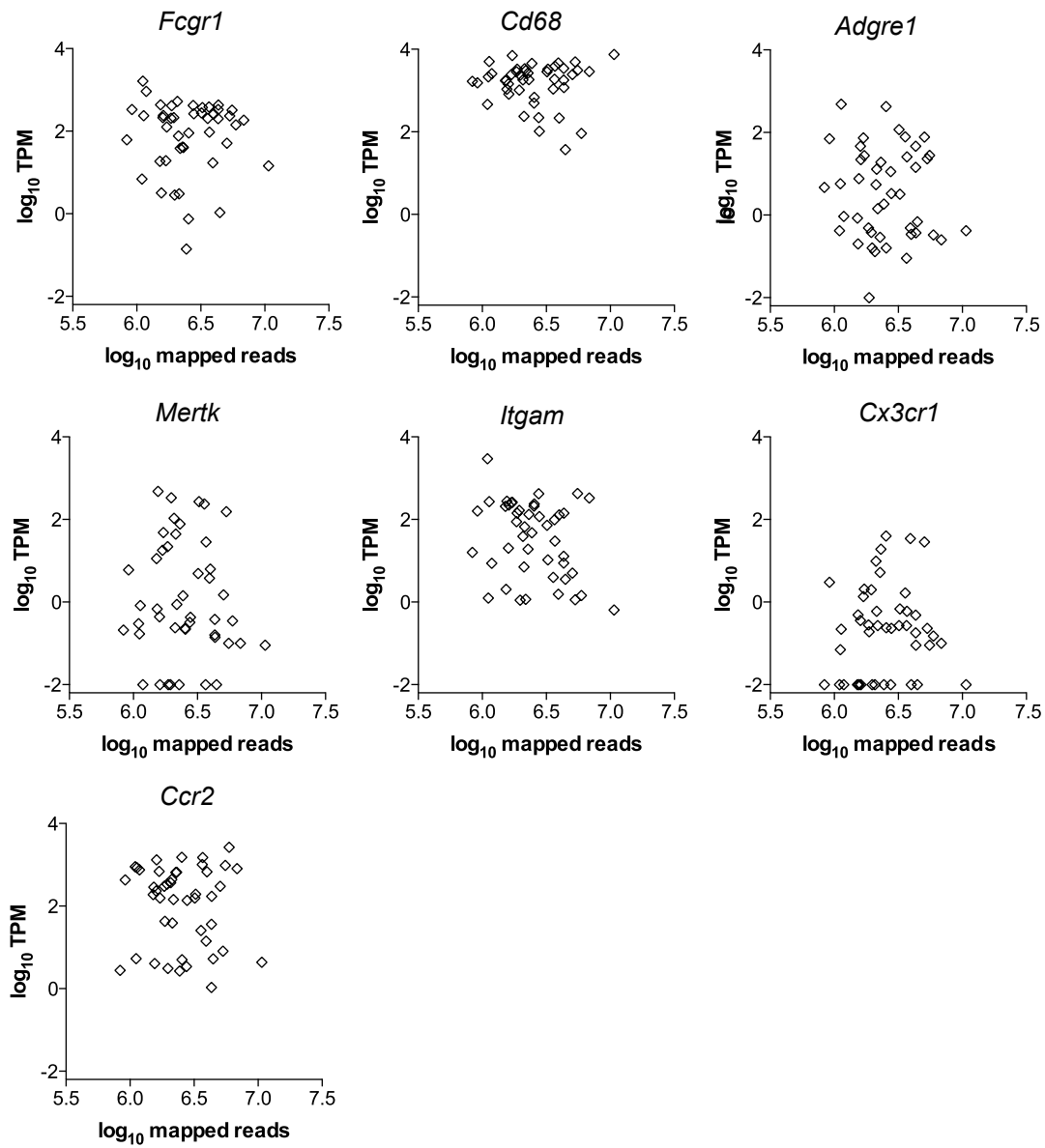

Supplement: Additional file 2: Figure S2. — Expression of myeloid lineage genes is not associated with read coverage. Expression (log10 TPM) as a function of mapped read coverage does not reveal bias. Of particular note, cells with zero detected expression of a given marker do not correlate with read coverage. (PDF 283 kb) [file 12974_2016_581_MOESM2_ESM.pdf]

**Figure S3**

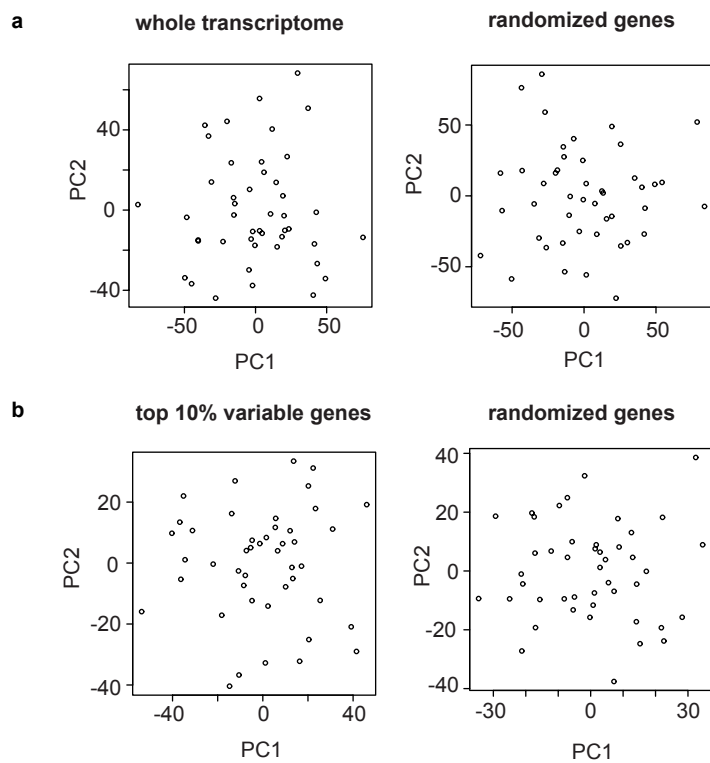

Supplement: Additional file 3: Figure S3. — Dimensionality reduction analysis of the whole transcriptome or of the top 10 % variable genes across all individual TBI macrophages shows a lack of coherent population structure. (a) PCA analysis of the whole transcriptome (38,126 genes). The same analysis applied to the same data, but in randomized order, is shown for comparison. (b) PCA analysis of the top 10 % of genes with the highest variance (3815 genes). Expression data from the same 3815 genes were randomly ordered and are shown for comparison. (PDF 166 kb) [file 12974_2016_581_MOESM3_ESM.pdf]

Figure S4

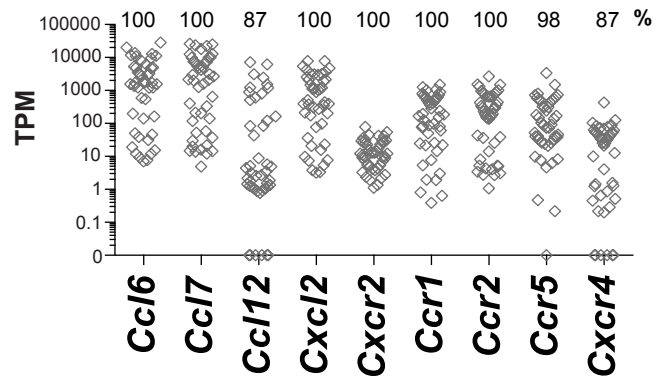

Supplement: Additional file 4: Figure S4. — Gene expression analysis of chemotaxis genes not associated with macrophage polarization. All or nearly all TBI macrophages express moderate to high levels of several chemotaxis-associated genes as shown. Each diamond symbol represents one TBI macrophage. The percentage of cells positively expressing a gene is shown above each gene’s column. (PDF 160 kb) [file 12974_2016_581_MOESM4_ESM.pdf]
